# Predatory bacteria can intensify lung-injury in a multidrug-resistant *Acinetobacter baumannii* pneumonia model in rat

**DOI:** 10.3389/fmicb.2025.1512119

**Published:** 2025-01-23

**Authors:** Zeinab Mohsenipour, Farzaneh Kianian, Behnaz Jahanbin, Hamid Reza Abtahi, Tooba Ghazanfari, Maryam Edalatifard, Saeid Amanpour, Mikael Skurnik, Parya Arazi, Mohammad Mehdi Feizabadi

**Affiliations:** ^1^Department of Microbiology, School of Medicine, Tehran University of Medical Sciences, Tehran, Iran; ^2^Department of Physiology, School of Medicine, Tehran University of Medical Sciences, Tehran, Iran; ^3^Department of Pathology, Cancer Institute, Imam Khomeini Hospital Complex, Tehran University of Medical Sciences, Tehran, Iran; ^4^Department of Pulmonary and Critical Care, Thoracic Research Center, Imam Khomeini Hospital Complex, Tehran University of Medical Sciences, Tehran, Iran; ^5^Immunoregulation Research Center, Shahed University, Tehran, Iran; ^6^Cancer Biology Research Center, Tehran University of Medical Sciences, Tehran, Iran; ^7^Department of Bacteriology and Immunology, Faculty of Medicine, University of Helsinki, Helsinki, Finland

**Keywords:** *Acinetobacter baumannii*, antibiotic resistant, *Bdellovibrio bacteriovorus*, predatory bacteria, respiratory tract infection

## Abstract

**Introduction:**

Respiratory tract infection caused by antibiotic-resistant bacteria are one of the most important causes of death worldwide. Therefore, in this study, we investigated the possibility of using predatory bacteria to improve the *Acinetobacter baumannii* pneumonia model in rat.

**Methods:**

Multidrug-resistant (MDR) *A. baumannii* clinical strain was used to induce pneumonia. In addition to the sham and predator control group, three treatment groups (*n* = 5) were studied with colistin, *Bdellovibrio bacteriovorus* HD100, and combination of predator and antibiotics. Also, the colistin MIC value for *B. bacteriovorus* HD100 (8 μg/mL) was determined for the first time to our knowledge. Removal of excess endotoxin from the predator suspension was performed with the help of organic solvents before inoculation of rats.

**Results:**

The most successful treatment was observed in the group treated with colistin followed by combined treatment. In the predator treatment group, the systemic spread of *A. baumannii* was lower than other treatment groups. However, treatment with predatory bacteria not only failed to reduce the pathogen load in the lungs to the same extent as the antibiotic treatment group, but also induced acute pulmonary and systemic inflammatory responses. Therefore, the rats showed the highest septic score (21.4 at 48 h) and did not survive more than 48 h.

**Discussion:**

This is the first report of systemic complications of using *B. bacteriovorus* HD100 for infection control. According to our results, the effects of predatory bacteria in the *in vivo* environment are complex and many questions need to be answered before it can be introduced as a live antibiotic.

## Graphical abstract

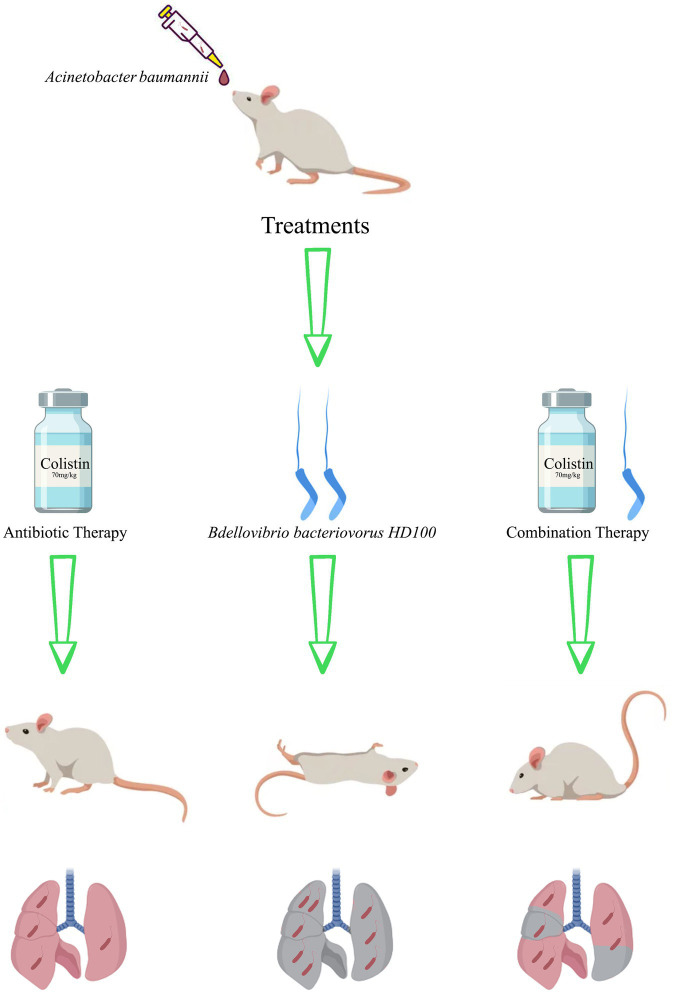

## Introduction

Pneumonia is the second most common hospital-acquired infection that ranks first among intensive care units (ICUs) patients. Hospital-acquired pneumonia (HAP) is often caused by drug-resistant bacteria and cause death in 20–70% of cases (Koulenti et al., 2017). In pneumonia, infection of the lung parenchyma is observed with acute inflammation and tissue consolidation. Therefore, the filling of bronchioles and alveoli with inflammatory secretions reduces gas exchange and causes respiratory symptoms. On the other hand, occurrence of severe inflammatory phase by immune cells in the lung is one of the important factors of tissue damage in pneumonia. Although this process is controlled by reducing the pathogenic agent under the process of immune homeostasis. However, infectious and some therapeutic agents, by interfering with homeostasis, intensify the inflammatory phase and cause tissue damage and disease progression (Gopallawa et al., 2023).

Gram-negative bacteria (GNB), especially Enterobacteria including *Klebsiella pneumoniae*, followed by *Acinetobacter baumannii*, and *Pseudomonas aeruginosa* are the most common bacterial causes of HAP (Torres et al., 2017). These bacteria are also part of the ESKAPE group (including *Enterococcus faecium*, *Staphylococcus aureus*, *K. pneumoniae*, *A. baumannii*, *P. aeruginosa*, and *Enterobacter* spp), and are on the priority list of the World Health Organization (WHO) to find new therapeutic methods due to their high drug resistance (Tacconelli et al., 2018). MDR (Multi-Drug Resistant) and XDR (Extensively-Drug Resistant) clinical isolates of *A. baumannii* constitute the dominant population of hospital-acquired infectious agents, and they cause high mortality, especially in systemic infections. Also, *A. baumannii* causes wide range of nosocomial infections such as pneumonia, bacteremia, meningitis, soft tissue infection, and urinary tract infection (UTI), which are seen especially in immunocompromised patients. Presence of different resistance mechanisms such as drug-degrading enzymes, efflux pumps and changes in the cell envelope have left limited antibiotic options for treatment of drug-resistant strains of *A. baumannii* (Ayoub Moubareck and Hammoudi Halat, 2020). Colistin is one of the last treatment choices for these strains, however, its toxicity and noticeable side effects limit its use (Zheng et al., 2020).

The presence of efflux pumps and their increased expression are among the most important mechanisms by which *A. baumannii* acquires resistance to antibiotics in particular to colistin. As a result of efflux pumps functions not being restricted to specific substrates, they participate in pumping out multiple antibiotics from the cell and are associated with the development of MDR strains. Five families of efflux pumps are responsible for drug resistance in *A. baumannii*. The first group is the resistance nodulation division (RND) superfamily (AdeABC, AdeIKJ, AdeFGH), which associates with developing resistance to aminoglycosides, fluoroquinolones, tetracyclines and carbapenems (Shi et al., 2024). The second family is the ATP-binding cassette (ABC) superfamily (AdeABC, MsbA) which contributes to resistance against carbapenems and various disinfectants. The third family is the small multidrug resistance (SMR) protein subfamily (AbeS), which is involved in resistance development to fluoroquinolones and macrolides. The fourth family is the multidrug and toxic compound extrusion (MATE) superfamily (AbeM), which causes resistance to sulfonamides, aminoglycosides and chloramphenicol by affecting H^+^ exchange. The last family is the major facilitator (MFS) superfamily (TetA, TetY, CraA, AmvA), which is involved in resistance development to tetracyclines, carbapenems and aminoglycosides (Chang et al., 2016).

Several methods for drug-resistant strains of *A. baumannii* infection control have been investigated, such as antibiotic combination therapy, herbal derivatives and phage therapy (Karakonstantis et al., 2021). Six decades after the discovery of the first predatory bacterium*, Bdellovibrio bacteriovorus*, studies on the use of predators as a live antibiotic in the control of drug-resistant pathogens continue. Predators are driven to prey-rich areas by chemotaxis, and then they get attached to the prey. Despite details of prey tropism still being vague, the role of type IV pili in initial attachment, rose-like structures, MIDAS adhesin family protein, and fiber-like proteins (mosaic adhesion proteins or MAT) on the surface of *Bdellovibrio* for prey attachment and subsequent entry into the periplasm are confirmed (Caulton et al., 2024; Tyson et al., 2024). Rose-like structures are only seen on the surface of predator cells during the attack phase (Huwiler et al., 2023). Also, MATs are a superfamily with diverse binding domains which let them bind to different cell envelopes of prey bacteria, and make predation possible on a wide range of preys (Caulton et al., 2024).

*Bedlovibrio* reproduces by entering the periplasm of the bacterial prey and then exits by lysing the host cell. The number of predators produced varies depending on the size of the prey cells and the amount of nutrients available for *Bdellovibrio* (Summers and Kreft, 2022). Effect on a wide range of gram-negative bacteria, nontoxic to mammalian cells (Gupta et al., 2016), and isolated *B. bacteriovorus* from the human gut (Iebba et al., 2013) are the reasons that make predatory bacteria an attractive option for combating pathogens. Limited *in vivo* studies have been performed with predatory bacteria. The aim of these studies has been to control local infections, and their results confirm the ability of predatory bacteria to reduce the pathogen’s load (Romanowski et al., 2016, 2023; Cui et al., 2021; Liu et al., 2023; Tajabadi et al., 2023). Furthermore, a significant systemic pathogen inhibition by *B. bacterivorus* has been demonstrated only in a few studies, i.e., oral administration to chickens (Atterbury et al., 2011), intranasal administration to rats (Shatzkes et al., 2016), and intravenous administration to rats (Shatzkes et al., 2017) and mice (Findlay et al., 2019).

A successful treatment should have limited and controllable side effects in addition to inhibiting the pathogen. Although the presence of modified lipid A in the LPS of *B. bacteriovorus* has been confirmed to weakly induce inflammatory cytokines (Needham and Trent, 2013), an important question is how long the continuous release of endotoxin from the prey pathogen can be tolerated by the host in a systemic infection? Perhaps the perpetual cycles of predation could be controlled using engineered predator bacteria privileged with specific prey preferences. Thus far, a single study has reported the effects of nasal administration of predatory bacteria to control *K. pneumoniae* in an experimental respiratory infection of rats (Shatzkes et al., 2016).

In the present study, we developed a rat pneumonia model with MDR clinical isolate of *A. baumannii* and used intratracheal administration of *B. bacteriovorus* HD 100 for treatment. Tissue tropism of the predatory bacteria, the time of their elimination from the host body, and the induction of inflammatory and destructive responses are important questions to be answered upon systemic use of a live bacteria as an alternative to antibiotics. To elucidate these questions, we studied the systemic release of predatory and pathogenic bacteria in the rat body and the response of two pro-inflammatory cytokines to the pathogen, predator and their interactions.

## Materials and methods

### Buffers, media and drugs

HEPES buffer: 6 g/L HEPES powder, 0.254 g/L MgCl_2_, 0.284 g/L CaCl_2_, pH 7.4; Top agar: HEPES buffer supplemented with 0.6% agar, pH 7.6; Bottom agar: same as Top agar supplemented with 1.5% agar, pH 7.6; 1-octanol (Merck, catalog number: 100991); Colistin (Sigma-Aldrich, catalog number: C4461), Colistimethate sodium (Exir, catalog number: 2205262/IRI); cyclophosphamide (CPM) (Baxter, catalog number: 23917472797); Ketamine (Bremer Pharma GmbH, catalog number: 30273), Xylazine (Serumwerk Bernburg AG, catalog number: QM03B).

### Bacterial strains and growth condition

A clinical MDR *A. baumannii* strain 256 previously isolated from COVID-19 patients was used as pathogen. This isolate was sensitive to colistin (Mobarak-Qamsari et al., 2023). Also, a colistin-resistant clinical isolate of *A. baumannii* was used as prey for antimicrobial susceptibility test of *B. bacteriovorus* strain HD100 (ATCC 15356) as predatory bacteria (Marine et al., 2020).

For antimicrobial susceptibility tests, colistin-resistant *A. baumannii* was grown in Nutrient broth (NB) media (Merck, Germany), and for co-culture preparation and *in vivo* tests, colistin-sensitive *A. baumannii* strain 256 was grown in Brain-Heart infusion (BHI) broth (Merck, Germany). *B. bacteriovorus* HD100 for all tests was prepared in a co-culture with a 3:1 prey: predator ratio in HEPES buffer that was incubated at 29°C with 180 rpm shaking for 48 h. Then, the co-culture was centrifuged (4000 rpm, 4°C, 10 min) and the supernatant was passed through a 0.45 μm filter to purify predatory cells. Finally, PFU (Plaque Forming Unit)/ml of *B. bacteriovorus* HD100 was determined by the double-layer agar technique (Starr, 1975).

### Antibiotic susceptibility test

The minimum inhibitory concentration (MIC) of colistin for *A. baumannii* strain 256 was determined in our previous study (20). Antimicrobial susceptibility of *B. bacteriovorus* HD100 to colistin was evaluated as previously described by Marine et al. (2020) with some modification. For MIC determination, growth of *B. bacteriovorus* HD100, *A. baumannii*, and also, co-culture of the prey and predator along with control wells were measured in the presence and absence of colistin. Briefly, two-fold serial dilutions of colistin in 100 μL HEPES buffer were prepared on 96-well microplates. Then, 50 μL of an over-night culture of colistin-resistant *A. baumannii* suspension (with a turbidity equal to 1 McFarland) was added to all wells except to the predator control wells. Also, 50 μL aliquots of *B. bacteriovorus* HD100 achieved from a filtrated 48 h co-culture (diluted 1:3 with HEPES buffer to achieve appropriate of predator: prey ratio) were added to all wells except to *A. baumannii* control wells. To equalize the dilution, 50 μL of HEPES buffer was also added to the control wells containing only *A. baumannii*, and only *B. bacteriovorus* HD100. OD_600_ at zero time was recorded by Microplate reader ELX800TS (Bioteck, USA). The microplates were incubated for 24 h at 29°C with 180 rpm shaking and then OD_600_ was recorded again. Furthermore, the relationships between the OD changes and survival rates of prey and predator bacteria were confirmed by calculating the CFU (colony forming units)/ml of *A. baumannii* and PFU/ml of *B. bacteriovorus* HD100 (Swebocki, 2023). Incubation was not continued for more than 24 h because the poor aeration and accumulation of waste products inhibited the predator, and the antibiotic sensitivity results were not reliable.

The tests were done in triplicate. All absorbance values were subtracted from the respective control wells. The OD-antibiotic concentration diagrams for *A. baumannii* and the co-culture wells were drawn. The survival of *B. bacteriovorus* HD100 in each antibiotic concentration was plotted based on PFU at 24 h. Then, the MIC value of colistin for predatory bacteria was defined as the minimum concentration that made the viability of *A. baumannii* incubated with colistin, significantly different in the presence and absence of the predator. In addition, the growth inhibition percentage of *A. baumannii* was determined for different concentrations of antibiotics as well as *B. bacteriovorus* HD100 using the formula:

$\begin{array}{l} \%\mathrm{growth inhibitory}=\left(OD\;\mathrm{untreated}-OD\;\mathrm{test}\right)\\ /\left(OD\;\mathrm{untreated}-OD\;\mathrm{blank}\right)*100 \end{array}$ .

### Preparation of predatory bacteria for *in vivo* study

For *in vivo* experiments, prey lysate endotoxins were removed in predator suspension using 1-octanol extraction (Szermer-Olearnik and Boratyński, 2015). This process was used for the first time in co-culture of a predatory bacteria. Briefly, MgCl_2_ was added to the filtered, co-cultured predatory bacteria to the final concentration of 0.02 M and kept at 4°C for 24 h. Then 1-octanol was added to 20% (vol/vol) and shaken at room temperature for 2 h at 180 rpm. The mixture was cooled to 4°C for 2 h and then the aqueous phase was separated by centrifugation (4,000 *g*, 4°C, 10 min). The aqueous phase was dialyzed against 15–20% ethanol (5 × 4 h) and then against 0.15 M NaCl (4 × 4 h).

For more efficiency and depending on the co-culture conditions, the process was repeated 2–3 times. Finally, the number of viable predatory bacteria in the detoxified suspension was calculated by determining PFU (Starr, 1975). Due to the presence of LPS in the predatory bacteria, the LAL (limulus amebocyte lysate) test was not a suitable method to ensure acceptable removal of endotoxin. For this reason, the effectiveness of the method was confirmed by intratracheal administration of detoxified suspension (*n* = 5/group) versus non-detoxified suspension (*n* = 5/group) to healthy rat and observation of septic score during 3 days (Sulzbacher et al., 2022).

### Animals and ethics

Healthy pathogen-free female Wistar rats bred at the Laboratory Animals Center of the Physiology Department of Tehran University of Medical Sciences (TUMS) were used. Rats (age, 12–16 weeks; body weight, 185–220 g) were randomly divided into experimental groups and housed separately in well-ventilated cages with free access to food and water, 18–25°C, and 12 h/12 h light/dark cycle. All animal experimental protocols of the present study were approved by the Ethics and Welfare Committee of Laboratory Animals of Tehran University of Medical Sciences (IR.TUMS.AEC.1401.120). All animal procedures were performed according to approved guidelines.

### Rat model of pneumonia

Transient neutropenia was induced by intraperitoneal injection of CPM (150 mg/kg) 1 day before inoculation. All rats were anesthetized with a mixture of ketamine (87 mg/kg) and xylazine (13 mg/kg). Then, rats were suspended by their upper teeth from a nylon thread on the angled intubation bed. 100 μL of *A. baumannii* strain 256 suspension (1.03 × 10^9^ CFU/mL) in sterile saline was gently introduced into the trachea through a 2.5 cm delivery tube, followed by instillation of air bolus with a sterile syringe (Bergamini et al., 2021).

### Study design and treatment plans

Rats were randomized into three treatment groups (*n* = 25/group), a sham group (*n* = 25), and three control groups, including non-infected healthy rats (*n* = 5), the *A. baumannii*-only infected (*n* = 25), and the predatory bacteria control rats (*n* = 25). Animals in the treatment groups were inoculated with 100 μL of *B. bacteriovorus* HD100 containing 1 × 10^8^ PFU/mL (predator therapy), 70 μL colistimethate sodium (20 mg/kg) (Ku et al., 2019) (antibiotic therapy), and combination of 100 μL predator and 70 μL antibiotic (combination therapy). The sham group was developed to control possible side effects of cyclophosphamide, and rats received only 100 μL of sterile saline. All treatment were administrated intratracheally. The predatory bacteria and antibiotic therapy were administered 30 min after *A. baumannii* inoculation (Shatzkes et al., 2016).

### Animal survival and bacterial clearance

For all animals in each group, daily weight, body temperature, and disease symptoms were recorded according to septic criteria (Sulzbacher et al., 2022). Also, the death rate in each group was recorded. Five rats in each experimental group were sacrificed at 30 min, 6 h, 24 h, 48 h, and 10 days post-inoculation. Whole blood was collected for white blood cell (WBC) count test (SYSMEX XS-500i). Furthermore, lung, liver, spleen, kidney, and brain were harvested for quantitative bacteriological studies. Tissue sampling was done under aseptic conditions. All tools were sterilized before starting work. Also, for prevent tissue contamination, first the surface of the animal’s body was sterilized with 70% alcohol and then sampling was done. Tissues were homogenized (Ultra-Turrax homogenizer, IKA, Germany) in 2 mL of sterile phosphate buffered saline (PBS). 20 μL serial 10-fold dilutions of homogenates were plated on BHI agar and incubated at 37°C for 24 h. Also, 100 μL serial tenfold dilutions of homogenates were used in double-layer agar technique to determine *B. bacteriovorus* HD100 PFU/mL.

### Histological examination

In all groups, the left lungs of rats were harvested and preserved in 10% formalin. The samples were dehydrated in 70% ethanol and embedded in paraffin. Finally, the 3.5-μm-thick sections of the specimen were stained with standard hematoxylin and eosin (H&E) protocol and observed with a light microscope (Morello et al., 2011).

### Measurement of proinflammatory cytokines

Blood samples were collected from anesthetized rats through cardiac puncture. Clotted blood was centrifuged at 4000 rpm for 20 min. Serum was collected and stored at −70°C for cytokine assays. The levels of two pro-inflammatory cytokines, TNF-*α* and IL-1β, in sera were measured by Rat TNF-alpha ELISA Kit and Rat IL-1 beta ELISA Kit (Zellbio GmbH, Germany), respectively.

### Statistical analysis

Graphs and statistical analyses were performed using GraphPad Prism 9.5. Significant differences were confirmed when *p* < 0.05. Data sets were presented as means ± standard deviations (SD) for each group. Differences in quantitative measurements were determined by two-way analysis of variance (ANOVA) followed by Tukey’s post-hoc tests. Also, Kaplan–Meier survival graph of rats was plotted.

## Results

### Antibiotic susceptibility test

**Figure 1:**
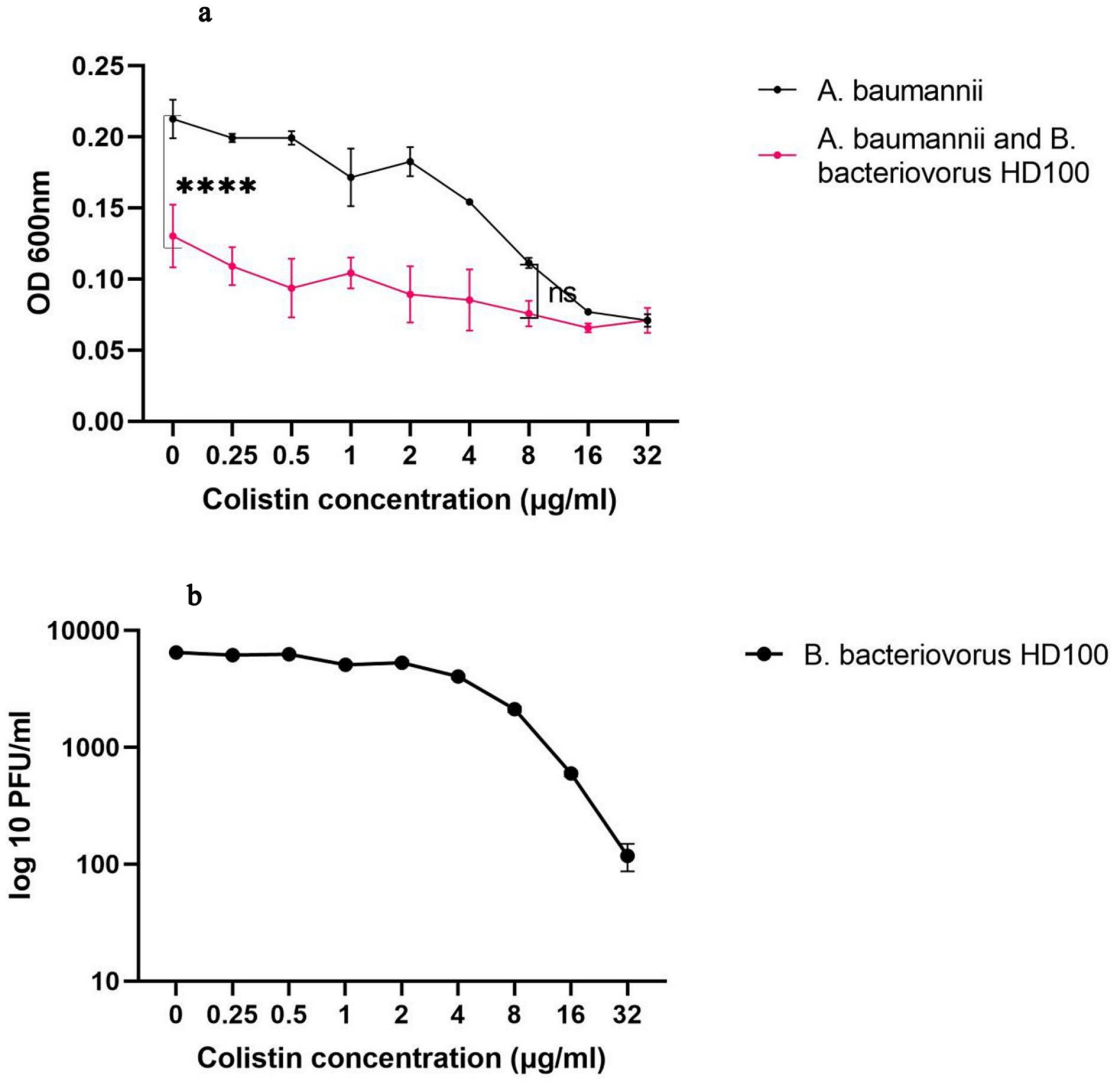
The colistin susceptibility determination for the predatory bacteria **(A)** OD-antibiotic concentration diagram for *A. baumannii* and co-culture of *A. baumannii* and *B. bacteriovorus* HD100. At a colistin concentration below 8 μg/mL the presence of the predator reduced the prey concentration significantly (****p-value <0.0001, ns: non-significant). **(B)** PFU/ml of *B. bacteriovorus* HD100 in the presence of different colistin concentration.

Since the growth of *B. bacteriovorus* HD100 *in vitro* necessarily requires prey bacteria, the sensitivity of the predator to antibiotics should be measured in a co-culture. On the other hand, due to the small size of the predatory bacteria, it does not cause significant light scattering at 600 nm wavelength. Both antibiotics and *B. bacteriovorus* HD100 decrease media opacity by inhibiting *A. baumannii* growth, complicating OD_600_ interpretation for predator activity. The optimal method for antibiotic susceptibility determination for predatory bacteria was to calculate the rate of prey inhibition in a co-culture compared to pure prey culture. The comparisons were carried out in the presence of different concentrations of antibiotics (0.25–32 μg/mL), and thereby it was possible to determine the contribution of the predator and the antibiotic in inhibiting the prey bacteria (Table 1). Therefore, we considered 8 μg/mL of colistin as the MIC of *B. bacteriovorus* HD100 as it was the minimum concentration that did not cause significant inhibition of *A. baumannii* growth in the presence of the antibiotic when compared to the co-culture with the antibiotic (Figure 1A). The survival of the predator was confirmed after 24 h in the wells by determining its PFU/mL (Figure 1B). These results confirmed that combination therapy of colistin and *B. bacteriovorus* HD100 in the animal model does not eliminate the predatory bacteria.

**Table 1:** Inhibition percentage of *A. baumannii* by antibiotic in the absences and presence of *B. bacteriovorus* HD100.

Colistin concentration (μg/mL)	% inhibition by antibiotic	% inhibition in co-culture	% predator efficiency
0	0	44.77%	44.77%
0.25	3.94%	57.40%	53.45%
0.5	3.94%	66.47%	62.52%
1	23.08%	60.16%	37.08%
2	13.81%	69.03%	55.23%
4	30.57%	71.40%	40.83%
8	56.02%	77.12%	21.10%
16	76.33%	83.04%	6.71%
32	79.88%	79.88%	0

### The endotoxin removal efficiency

Comparing the results of the double-layer agar test before and after endotoxin removal showed that although the numbers of predatory bacteria were reduced during this process (Appendix Figure 1A), they survived in acceptable numbers with the mean count 9.21 × 10^8^ PFU/mL during the tested 5 days. If the animal group was administered by non-treated *B. bacteriovorus* HD100 suspension, no rats survived more than a day. On the other hand, administration of the endotoxin-deprived predator suspension caused no death of animals before the fifth day (Appendix Figure 1B). Also, the septic score was significantly higher in animals administered with the non- treated suspension compared to endotoxin- deprived suspension (Appendix Figure 1C).

### Host mortality and septic score

**Figure 2:**
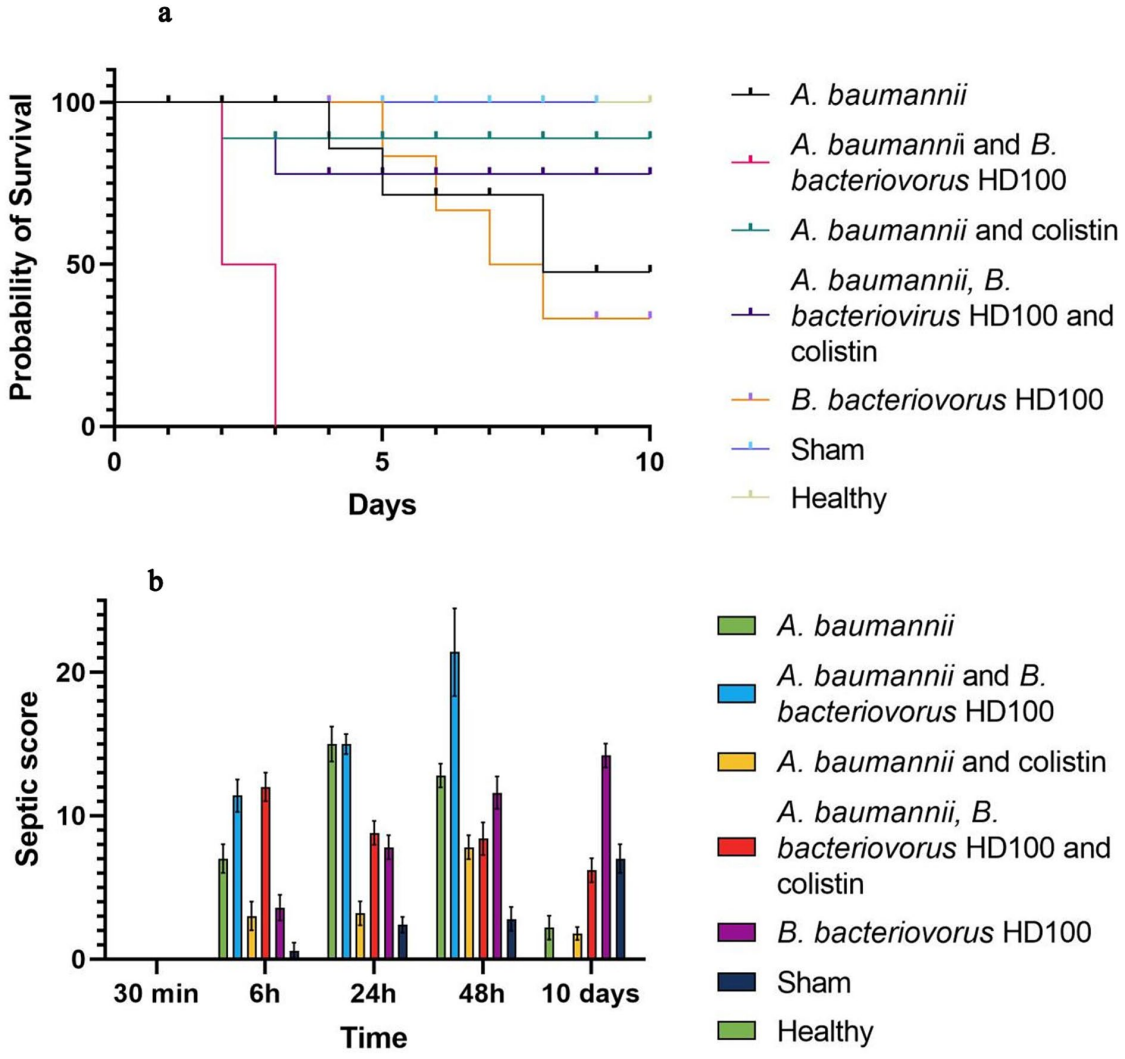
Host survival and septic score **(A)** survival curves of each group in 10 days based Kaplan–Meier analysis **(B)** septic score of each group during 30 min to 10 days.

The survival plots and septic score of all groups are shown in Figure 2. During 10 days, no deaths were observed in the healthy control and sham groups. The percentage of survival on day 10 was 50% for the infected control group, 90% for the colistin treatment, 80% for the combined antibiotic and predator treatment group, and 40% for the *B. bacteriovorus* HD100 control group. None of the animals treated with predatory bacteria survived more than 48 h (Figure 2A).

Based on the septic criteria and daily observations, the septic score was determined for all groups at 30 min, 6 h, 24 h, and up to 10 days (Figure 2B). The highest septic score in 6 to 48 h was related to the group treated with *B. bacteriovorus* HD100, the maximum of which was observed with a mean score of 21.4 at 48 h. Also, the lowest score in this period of time was for the sham group, and no signs of infection were observed in the healthy group. On day 10, the infection score of the sham group increased (mean score 7), and the highest score was observed for the control group of predatory bacteria (mean score 14.2).

### Pathogen clearance

**Figure 3:**
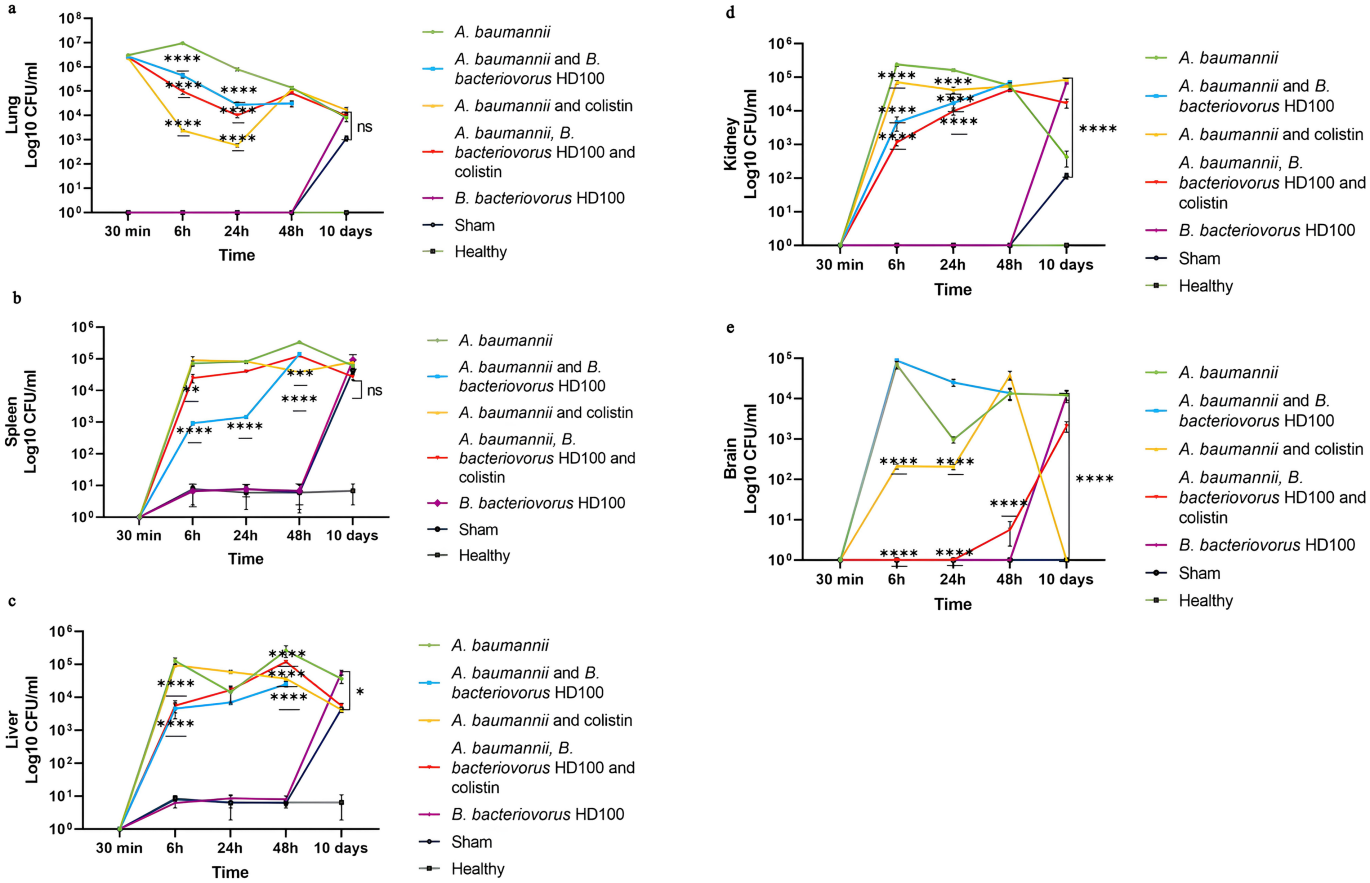
*A. baumannii* counts for each group between 30 min to 10 days **(A)** lungs, **(B)** spleen, **(C)** liver, **(D)** kidney, and **(E)** brains. Significancy only showed for the decrease in the comparison with infection and treatment groups (****p-value <0.0001, ***p-value <0.001, **p-value <0.01).

Figure 3 shows the bacterial pathogen load in five organs in each group for 10 days. MDR *A. baumannii* spread rapidly in the lungs and to the studied tissues. In the infection control group, within 30 min after inoculation, the average bacterial counts for the lungs were 3.05 × 10^6^ CFU/mL. The lung counts were highest at 6 h (9.5 × 10^6^ CFU/mL) and decreased over time, indicating persistent infection. The lung counts showed significant decreased (*p*-value <0.0001) in all three treatment groups only at 6 and 24 h post-inoculation (Figure 3A). The highest pathogen clearance in lung during the first 24 h post-inoculation was achieved by the antibiotic treatment, followed by the combined treatment, and the predator treatment, with mean counts of 1.5 × 10^3^, 5.36 × 10^4^, and 2.36 × 10^5^ CFU/mL, respectively.

In the *B. bacteriovorus* HD100 treated group, the pathogen bacterial counts in spleen, liver, and kidney decreased significantly (*p*-value <0.0001). Colistin treatment also reduced pathogen counts in the kidney, brain, spleen, and liver (Figures 3B–D). The predatory bacteria control group showed a significant number of microbes in the homogenized organs on day 10. A similar pattern was observed for the sham group, although no bacteria were isolated from the brain samples. Also, the microbial load of liver, kidney, and brain in the sham group was lower compared to the control group of predatory bacteria (Figures 3C–E).

### Predator distribution

Appendix Figure 2 presents the predator microbial load in different organs of each experimental group. No plaques were observed in double-layer agar tests from organs of rats not inoculated by *B. bacteriovorus* HD100. At 6 h after inoculation, the count of predatory bacteria in all organs (except the brain) in the *B. bacteriovorus* HD100 control group was significantly higher than in the group treated with the predator and combined treatment with antibiotics (*p*-value <0.0001). The number of predators in the brain at 6 h for treatments with *B. bacteriovorus* HD100, and combined treatment (1.07 × 10^3^ and 1.18 × 10^3^ PFU/mL, respectively) was much higher than the control group (7.96 × 10 PFU/mL). This pattern was observed in all organs (except kidney) at 24 h post-inoculation (Appendix Figure 2).

At 48 h, the number of predators in the combined treatment group was significantly lower than the control group (*p*-value <0.0001). On day 10 post- inoculation, this pattern was repeated for all organs (except kidney). On the other hand, at 48 h post-inoculation, the group treated with *B. bacteriovorus* HD100 showed a significant difference with the control group only in the liver (5.64 × 10^4^ PFU/mL versus 3.41 × 10^4^ PFU/mL for the control group) and kidney (2.46 × 10^4^ PFU/ml versus 1.47 × 10^4^ PFU/mL for the control group).

### Lung histopathology

**Figure 4:**
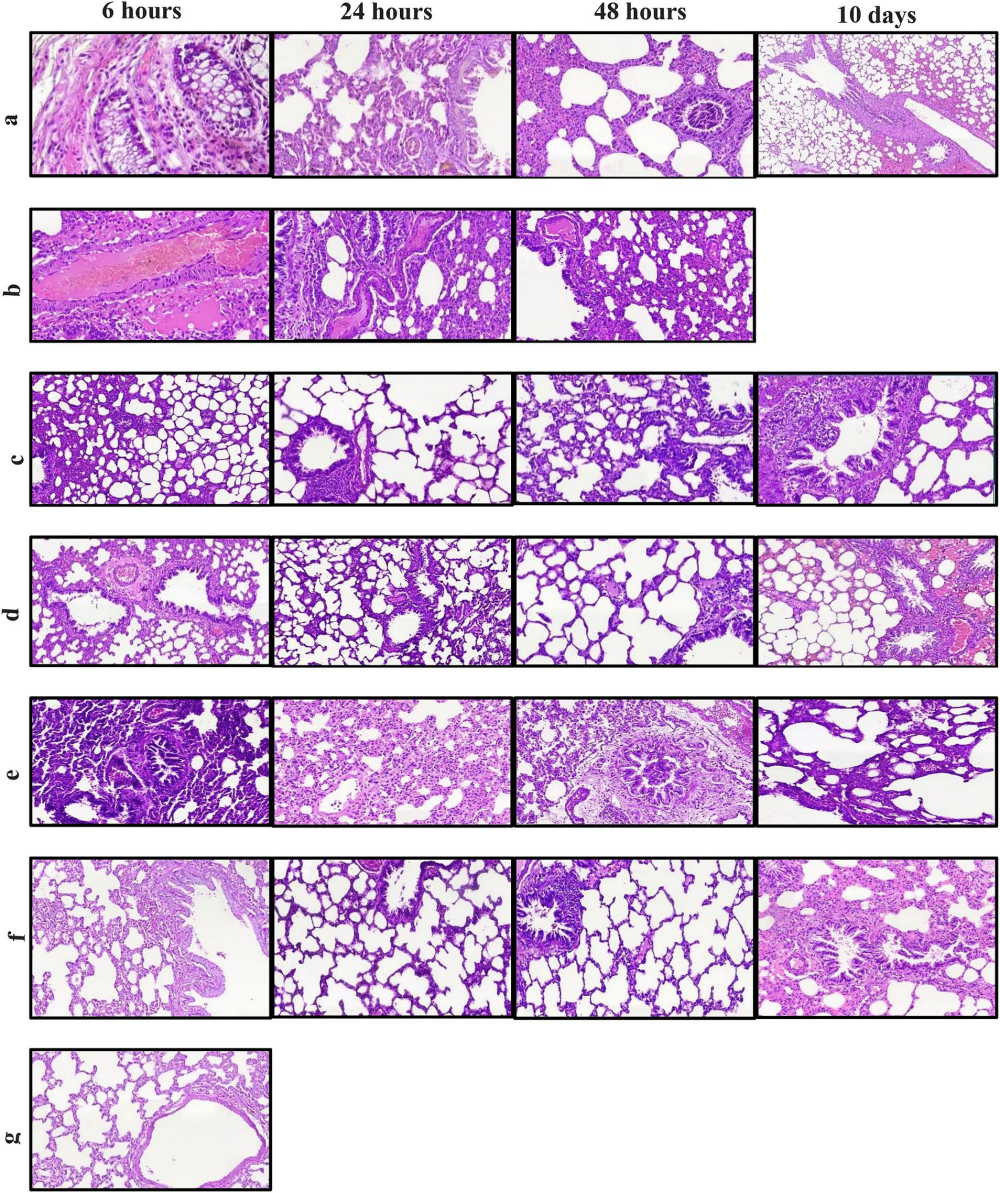
Hematoxylin and eosin staining of lung sections for each treatment groups between 6 h and 10 days. **(A)** infection control group **(B)** *B. bacteriovorus* HD100 treatment group, **(C)** colistin treatment group, **(D)** colistin and *B. bacteriovorus* HD100 combination treatment group, **(E)** *B. bacteriovorus* HD100 control group, **(F)** sham group, and **(G)** healthy group.

Acute neutrophil infiltration in alveoli from 6 h after pathogen inoculation confirms acute pneumonia in the infection control group (Figure 4A). The mixed inflammation mostly with the presence of neutrophils in this group continued for 48 h, but with time, alveolar involvement decreased, indicating clearance of the pathogen, and therefore the animal survived up to 10 days post-infection. Inflammatory response in the lung was patchy in all groups, but in *B. bacteriovorus* HD100 treatment group, inflammatory involvement was observed in most of the lung tissue, which was accompanied by abscess formation. In this group, in 6 h to 48 h, severe inflammatory responses with the presence of high neutrophils and involvement of lung parenchyma were observed (Figure 4B). Therefore, the animals of this group did not survive more than 48 h.

The antibiotic treatment group exhibited an initial acute inflammatory reaction, followed by temporary relief at 24 h but mild neutrophilic inflammation at 48 h (Figure 4C). In the combined treatment group, less inflammation was seen at 6 h compared to the antibiotic treated group, persisting to a lesser extent at 24 and 48 h (Figure 4D). The predatory bacteria control group displayed moderate to severe inflammation, with lymphocytic involvement persisting up to day 10 (Figure 4E). The healthy and sham groups did not show significant histologic abnormalities (Figures 4F, G).

### Systemic inflammatory response

**Figure 5:**
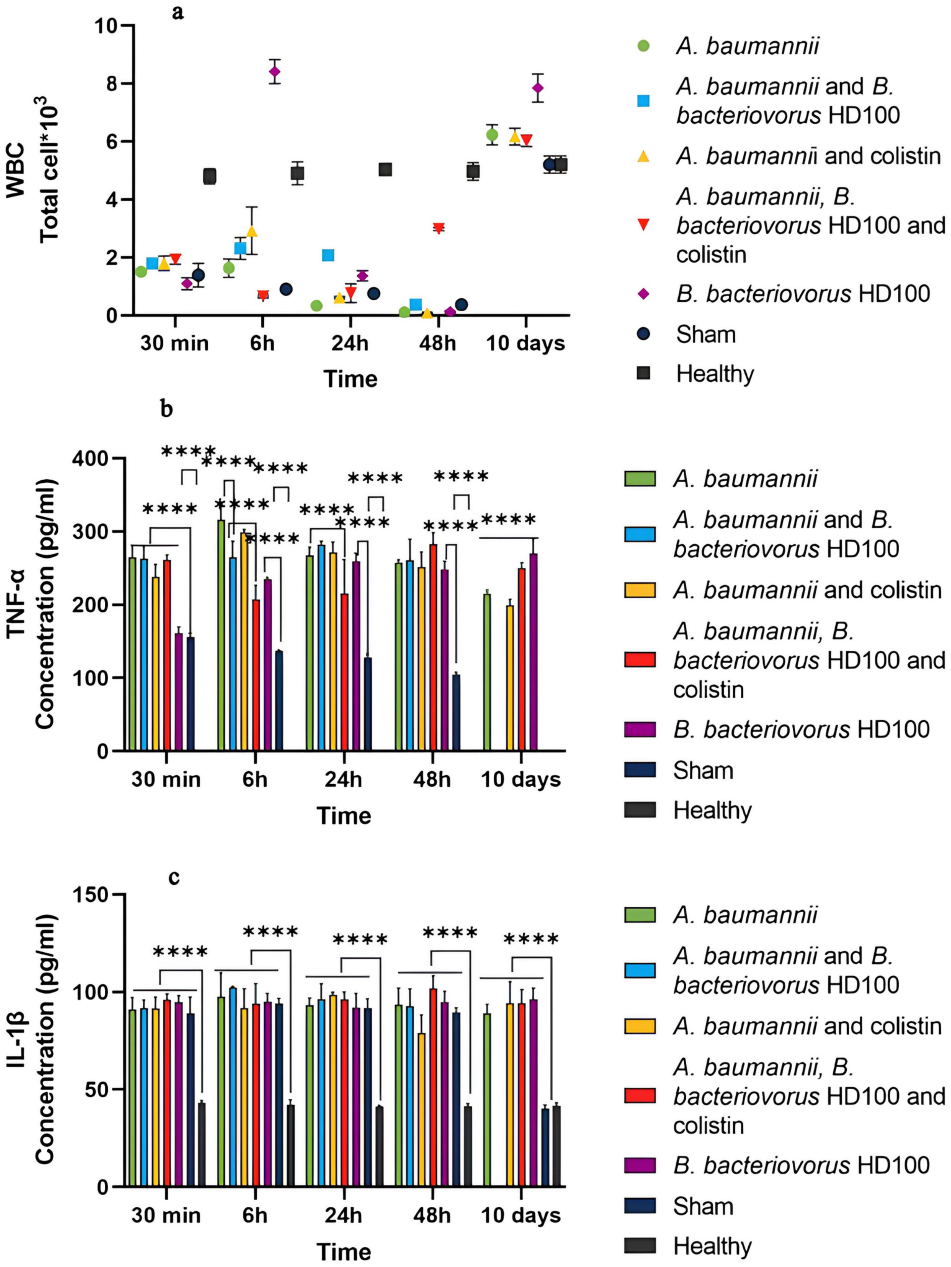
**(A)** Total white blood cell (WBC) counts for each group during the time of 30 min to 10 days. WBC counts using the SYSMEX XS-500i hemocytometer. TNF-*α* levels in the sera, **(B)** and IL-1β **(C)** levels in the sera of each group during the time of 30 min to 10 days. Significant differences are shown with the sham group as well as between the treatment and infection control groups (****p-value <0.0001).

Figure 5 shows the results of WBC counts for each group. About 18 h after CPM injection (30 min after treatment administration), the WBC levels in all groups (with the mean count of 1.58 × 10^3^ cells/mL) had decreased when compared to the healthy control group (4.8 × 10^3^ cell/mL). The WBC decrease for the sham group continued until 48 h (0.37 × 10^3^ cell), but on day 10, the WBC level returned to normal. On the other hand, the WBC levels increased at 6 h post-treatment for all treatment groups except for the combined treatment group, indicating acute inflammation. The *B. bacteriovorus* HD100 treatment group showed a decrease at 48 h. By day 10, WBC counts had increased in all groups, and the predator control group showing the highest increase. The detailed percentages of different cell types for each WBC are shown in Appendix Figures 3A–E.

In the sham group, the level of both TNF-*α* and IL-1β showed a significant increase from 30 min (about 18 h after CPM injection) to 48 h compared to the healthy group, but it was in the normal range on day 10 (Figures 5B,C).

The level of TNF-α was not different between the treatment groups at 30 min, but there was a significant difference in the predator control group and the sham group (Figure 5B). Also, the cytokine levels were significantly different between 6 h and 10 days with the sham group, which confirms the inflammation caused by infection and treatment. At 6 h, TNF-α level of the group treated with predatory bacteria (264.545 pg/mL) and the combined treatment (207.44 pg/mL) was significantly lower than that of the infectious control group (315.75 pg/mL). While antibiotic treatment (298.78 pg/mL) did not show such a difference. At 24 h, only the combination treatment (215.26 pg/mL) effectively reduced TNF-α compared to the infected control group (267.27 pg/mL). At 48 h and 10 days, the level of TNF-α in the treatment groups was equal to or higher than the infectious control group. The level of TNF-α for the *B. bacteriovorus* HD100 control group was significantly higher than the sham group from 6 h to day 10, and the highest on day 10 (270.30 pg/mL).

The level of IL-1β for all the tested groups in the period of 30 min to 48 h was not significantly different from each other and the sham group, but it was significantly higher than the healthy group. Also, this difference was present on the tenth day compared to the healthy and sham groups (Figure 5C).

## Discussion

The increase of antibiotic resistance in *A. baumannii*, which is one of the common causes of HAP, shows the necessity of finding an alternative to deal with this pathogen (Ayoub Moubareck and Hammoudi Halat, 2020). Therefore, in this study, we established an acute pneumonia model with an MDR strain of *A. baumannii* and evaluated the efficiency of the *B. bacteriovorus* HD100 as a novel treatment agent. Because *A. baumannii* is an opportunistic pathogen, acute pneumonia was induced in Wistar rats by inducing transient neutropenia, inoculation with a high dose, and using a clinical *A. baumannii* isolate. *A. baumannii* spread throughout the animal’s lung within 30 min after intratracheal administration (Figure 3), and the pathogen load in lung increased up to 6 h, and then showed a continuous decrease. Similar rapid systemic spread of *A. baumannii* has been reported in mice and has been attributed to the escape of the pathogen from the host’s innate immune system (Harris et al., 2019). Also, similar studies showed that the peak of the microbial counts of *A. baumannii* in the lung occurs between 4 h to 6 h after inoculation and then decreases. Furthermore, a higher pathogen load in lungs than other organs is indicative of a persistent lung infection (Russo et al., 2008; Harris et al., 2019).

According to the antibiogram results for clinical *A. baumannii* isolate, colistin was used for antibiotic treatment (Mobarak-Qamsari et al., 2023). CMS is administered intravenously or by inhalation and is degraded in body to active colistin, which has a half-life of approximately 4 h. Colistin efficacy and penetration are related to tissue characteristics such as tissue vascularity, lipid composition, extracellular matrix composition, tissue inflammation and drug binding affinity. Greater vascular muscle tissues lead to more facilitated colistin penetration compared with less vascular adipose tissues. Conversely, more fat in the tissue is associated with reduced drug penetration (Sanabria et al., 2021). Although colistin has a high electrostatic binding affinity for the lipid membranes of the liver, kidney, lung, brain, and heart; the drug penetration into the pleural cavity, lung tissue, cerebrospinal fluid, and bones is poor, and the concentrations are not considered antibacterial. In addition, increased inflammatory responses in the tissue are associated with reduced drug penetration and efficacy. Just like the increased-inflammation in patients with severe respiratory infections, inhaled administration of CMS has been recommended to achieve higher drug concentrations and reduce systemic side effects in these patients (Mondal et al., 2024). In addition, studies have confirmed that in the pulmonary administration, the plasma concentration of the drug remains high for a longer period of time (Yapa et al., 2013). Also, for combination therapy of predatory bacteria with colistin, we determined MIC to measure the sensitivity of *B. bacteriovorus* HD100 to colistin (8 μg/mL). Because MIC values in our study were not determined under the CLSI standard conditions, it cannot be compared with other bacteria. Also, for the first time, to our knowledge, *B. bacteriovorus* HD100 suspension for intratracheal administration was purified from endotoxins by the solvent-extraction method (Szermer-Olearnik and Boratyński, 2015).

The most effective treatment was observed in the antibiotic treated group and followed by combined treatment group. High survival rate and the lowest septic score during 10 days, the lowest lung bacterial counts up to 24 h, and the lung mild inflammatory response at 24 h and later were the benefits of the antibiotic treatment. However, a more intense infiltration of neutrophils at 6 h post-inoculation was observed for the antibiotic group than for the combined treatments. It is probably due to the infection of a large number of *A. baumannii* by the predator, and as a result, the antibiotic did not affect the pathogen. Therefore, less endotoxin was released and these groups also had a lower septic score and TNF-*α* serum level. On the other hand, the most severe pulmonary inflammatory reactions occurred in the *B. bacteriovorus* HD100 treated group, that continued up to 48 h. Studies that have used pulmonary administration of LPS to induce lung injury have reported similar pulmonary and systemic inflammatory responses to our observations. But these effects disappeared after 24 h, because they had used a single dose of endotoxin (Chuang et al., 2013; Fu et al., 2017).

Studies have shown that the most important early defense against *A. baumannii* respiratory infection is involved with macrophages and neutrophils cooperation. The release of LPS and its interaction with Toll like receptor-4 (TLR-4) activates M1 macrophages in the lung, which ultimately leads to the production of inflammatory cytokines and chemokines such as macrophage neutrophil chemokine-2 (MIP-2), IL- 1β, and TNF-α. Moreover, bacterial OMVs also play a role in activating the MyD88 pathway in the recruitment of immune cells by activating TLR-2. Therefore, large numbers of neutrophils are driven to the lung, which are primarily responsible for phagocytosis and killing of the pathogen and contribute to the recruitment of other immune cells through the TLR4-NFκB pathway (Marion et al., 2019). In the presence of large numbers of *A. baumannii*, more neutrophils are recruited to the lung, causing more inflammation and further damage to the airways (Harris et al., 2019). Since in lung histology, cell damage caused by the presence of the predator alone or together with the host pathogen has not been observed, the persistent inflammatory reactions in our study are probably due to the continuous release of endotoxin due to the continuous degradation of pathogens in the predation cycle. On the other hand, the three groups that received the predator showed continuous high levels of TNF-α after 6 h (except for the combined treatment at 24 h).

Even in the *B. bacteriovorus* HD100 control group, pulmonary inflammatory response and serum inflammatory cytokine levels were increased during the study period. So, the group showed the highest septic score and mortality rate on day 10. These observations may also suggest that the predatory bacteria may influence the microbiota modification. Since *B. bacteriovorus* HD100 is an obligate predator, it must have found a host in the body that can be dissected by day 10. In a study that examined the lung microbiome of cystic fibrosis (CF) patients for 1 year, the presence of predatory bacteria *Bdellovibrio* and *Vampirovibrio* was confirmed in the lungs. In addition, minor changes in the composition of the microbiome were observed, which was associated with the reduction of *Parcobacterium*. According to the curves presented in this manuscript, more predatory bacteria were isolated with higher disease severity (de Dios Caballero et al., 2017).

The lower systemic spread of *A. baumannii* can be considered as the only advantage of the treatment with predatory bacteria. This was also observed for combination therapy and is probably related to pathogen involvement in the lung in the predation cycle. A major problem in intratracheal inoculation of predatory bacteria was the rapid spread to the brain of the animals, where they persisted until day 10 of examination. Im et al. showed that *B. bacteriovorus* HD100 is resistant to serum complement system, which is likely related to the neutral charge of the predator LPS. Thus, as we observed in this study (Appendix Figures 2B–E), systemic spread of the predator is facilitated (Im et al., 2017). Similarly, Findlay et al. studied systemic administration of *B. bacteriovorus* HD100 to treat plague in mice. Although the predatory bacteria saved the mice from death and reduced the amount of the pathogen, they remained in the animal’s body for a long time. Also, imaging of the labeled predator revealed that *B. bacteriovorus* HD100 has tropism to fat-based tissues. The presented images confirmed the high intensity of the predator’s presence in the brain of the animals (Findlay et al., 2019).

*In vivo* systemic studies on predatory bacteria are very limited. In a study, Shatzkes et al. reported that intravenous administration of *B. bacteriovorus* has no effect on reducing the systemic burden of *K. pneumoniae* in rats. It also took 20 days for the predator to be removed from the animal’s body (Shatzkes et al., 2017). In another study by the same researchers, intranasal administration of *B. bacteriovorus* and *Micavibrio aeruginosavorus* was evaluated to reduce the microbial load of *K. pneumoniae* in the lung. The results showed that the lung pathogen load was reduced by more than 3.0 log10 in the animals, but no histological complications in the lungs or continuous increase of inflammatory cytokines were reported (16). These results may be related to use of immunocompetent rats and because *K. pneumoniae* is not a rat pathogen and is easily cleared by an efficient immune system. In addition, intranasal administration helps eliminate a number of pathogens and predatory bacteria before reaching the lungs. However, an acute inflammatory response in the lungs at 24 h after *B. bacteriovorus* HD100 inoculation was reported by these authors (Shatzkes et al., 2016).

It seems that the complications observed in our research are related to the continuous predatory cycle due to the wide range of prey, as well as, the wide systemic circulation of the predators. Therefore, the first step to use predatory bacteria as living antibiotics is to focus on the predation control through genetic modification. Despite the obscures life cycle of predatory bacteria and their interactions with the host, relying on new findings is promising. Studies have shown that about 40% of *B. bacteriovorus* genes, called the predatosome, are related to predation. Duncan et al. divided this set into four functional gene classes, of which class III is involved in bdelloplast turnover and predator evasion. Although, *B. bacteriovorus* causes prey cell death within 10–20 min after entry, it requires 3–4 h to complete the predation cycle. Genes involved in badloplast turnover and predator evasion are suitable candidates for gene manipulation to limit predation cycles (Duncan et al., 2019). Therefore, it is possible to engineer predatory bacteria that can enter the periplasm of the prey and destroy it while not being able to complete their cycle.

Another option is to alter proteins involved in prey binding. One example is the induction of mutations in the MAT complex, which can create predators with limited host range. However, due to the large number of MAT proteins, single-gene mutations can only delay predation (Caulton et al., 2024). Another suggestion is the induction of mutations in Bd0875, a protein of the MIDAS adhesin family, which was investigated in the study by Tyson et al. Mutations in this protein cause the formation of round and dead badloplasts (Tyson et al., 2024). It is also possible to generate engineered strains in which gene expression is controlled by riboswitches, as Dwidar and Yokobayashi did by generating theophylline-responsive strains that affected flagellar production and hunting ability in *B. bacteriovorus* (Dwidar and Yokobayashi, 2017).

## Conclusion

Determining the role of predatory bacteria on the microbiome and the consequences of these changes is an important question that needs to be answered. Furthermore, predator–prey interactions and their effects on immune responses in an *in vivo* setting should be evaluated. More research needs to determine when predatory bacteria are eliminated from the host’s body. Are predator metabolites responsible for inflammatory responses, or endotoxin, which is released from gram-negative prey once after each predator cycle, plays a role in inflammatory complications. Finally, considering the complications observed by predatory bacteria in our study, it can be said that there is still a long way to introduce these microbes as a live antibiotic. On the other hand, the results of this research can suggest a study on the possibility of predators in acute lung diseases. Therefore, controlling the population of predators in the lung microbiome of pneumonia patients may be possible to help them survive.

## Data Availability

The original contributions presented in the study are included in the article/supplementary material, further inquiries can be directed to the corresponding author.
