# Supplementary material for: Predatory bacteria can intensify lung-injury in a multidrug-resistant Acinetobacter baumannii pneumonia model in rat
Source: Front Microbiol. 2025 Jan 23;16:1512119. doi: 10.3389/fmicb.2025.1512119 (PMC11798975; doi:10.3389/fmicb.2025.1512119)
Supplement: Supplementary file 1 [file Data_Sheet_1.pdf]

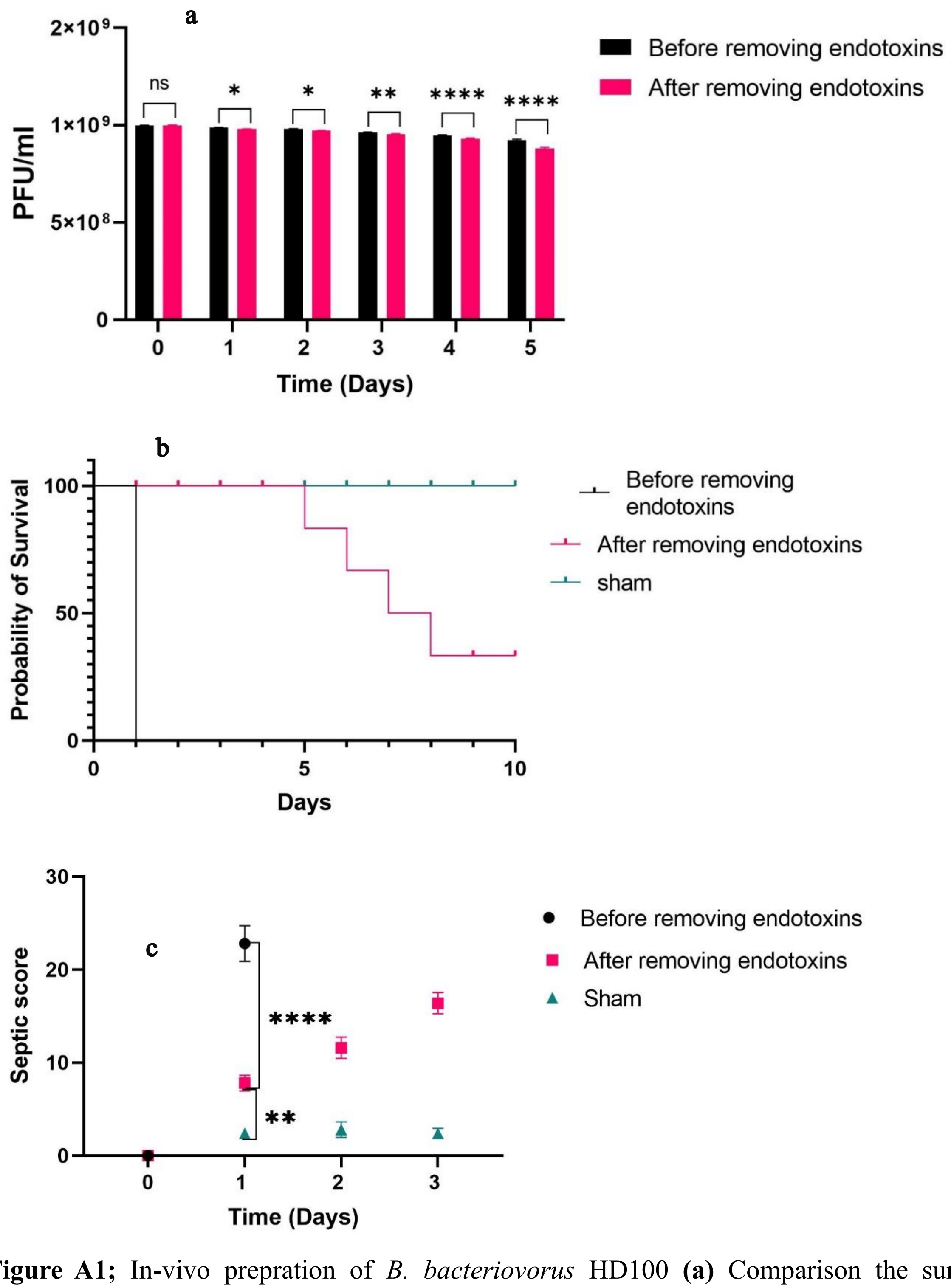

Figure A1; In-vivo prepration of *B. bacteriovorus* HD100 **(a)** Comparison the survival of predatory bacteria before and after removing endotoxin by determining PFU/ml **(b)** Survival of rats after intratracheal administration *B. bacteriovorus* HD100 with and without removing endotoxins **(c)** Septic score of animals treated with *B. bacteriovorus* HD100 with and without removing endotoxins. (****:P-value < 0.0001, **:P-value < 0.01, *:P-value < 0.05)

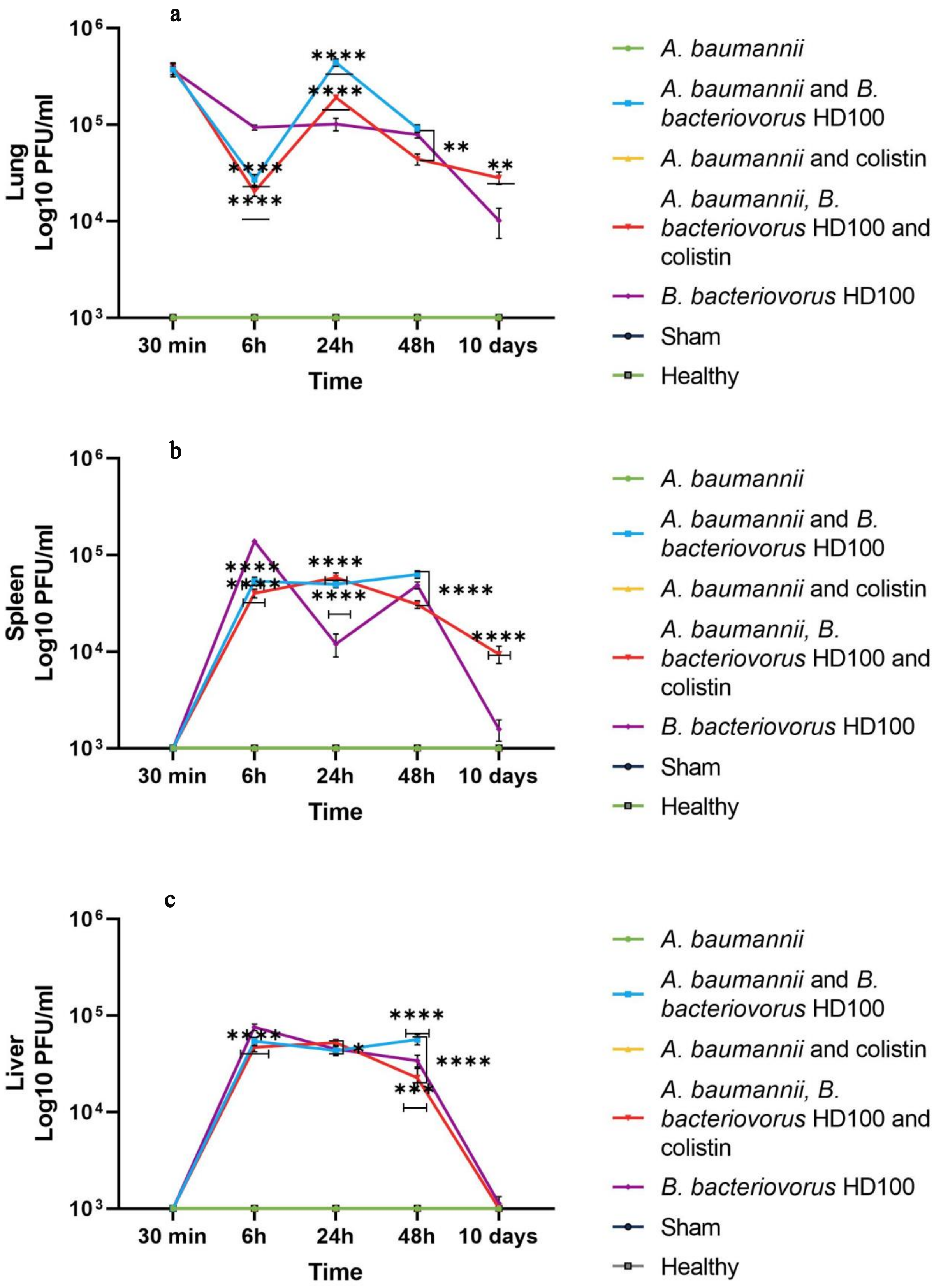

a
Lung
Log10 PFU/ml
10^6
10^5
10^4
10^3
30 min
6h
24h
48h
10 days
Time
****
****
****
****
**
**
A. baumannii
A. baumannii and B. bacteriovorus HD100
A. baumannii and colistin
A. baumannii, B. bacteriovorus HD100 and colistin
B. bacteriovorus HD100
Sham
Healthy
b
Spleen
Log10 PFU/ml
10^6
10^5
10^4
10^3
30 min
6h
24h
48h
10 days
Time
****
****
****
****
****
****
A. baumannii
A. baumannii and B. bacteriovorus HD100
A. baumannii and colistin
A. baumannii, B. bacteriovorus HD100 and colistin
B. bacteriovorus HD100
Sham
Healthy
c
Liver
Log10 PFU/ml
10^6
10^5
10^4
10^3
30 min
6h
24h
48h
10 days
Time
****
*
****
****
***
A. baumannii
A. baumannii and B. bacteriovorus HD100
A. baumannii and colistin
A. baumannii, B. bacteriovorus HD100 and colistin
B. bacteriovorus HD100
Sham
Healthy

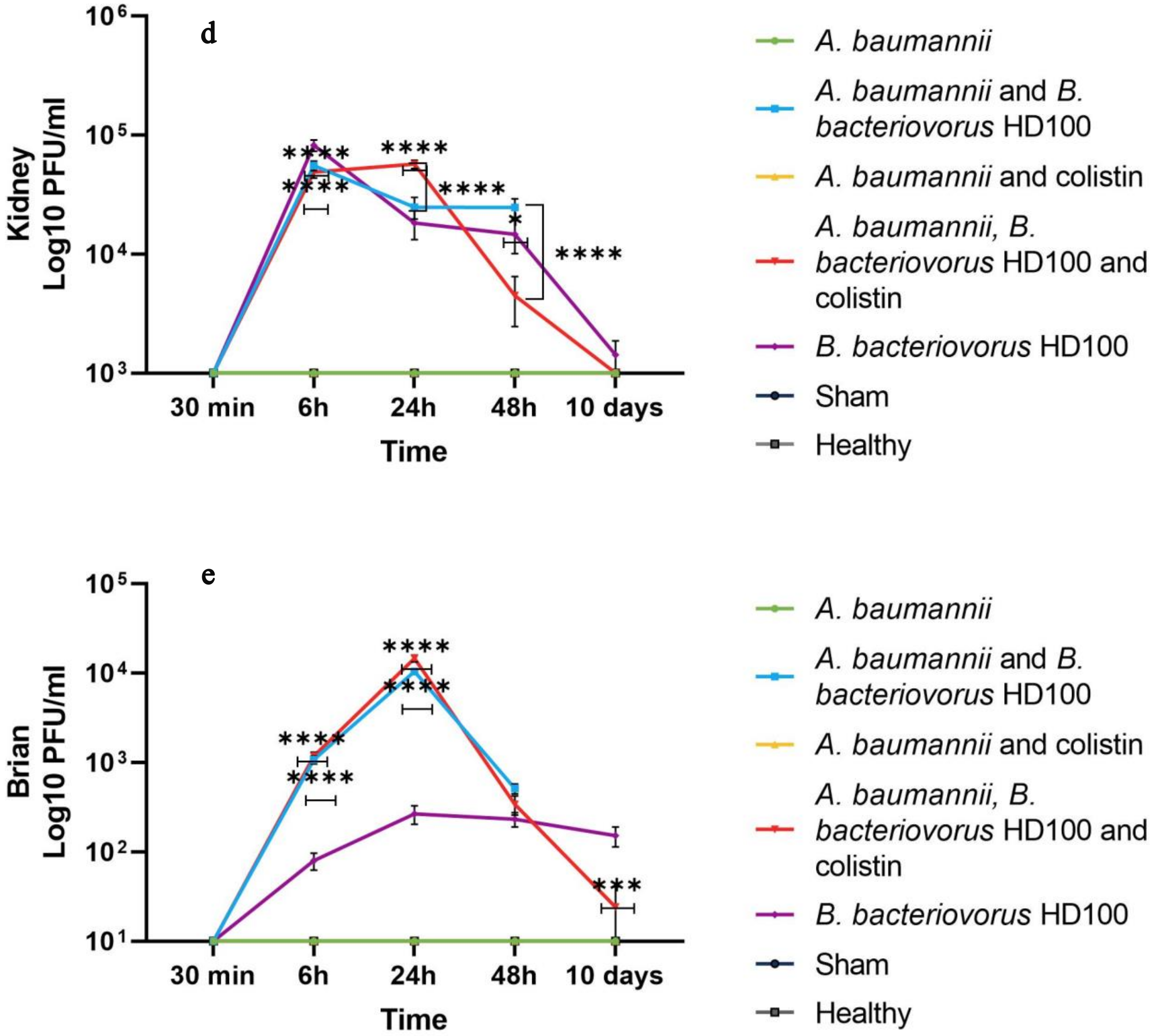

Figure A2. Load of *B. bacteriovorus* HD100 for each group between 30 minutes and 10 days. **(a)** lungs, **(b)** spleen, **(c)** liver, (d) kidney, and **(e)** brains. Significant differences are shown to the predator control group (****:P-value < 0.0001, ***:P-value < 0.001, **:P-value < 0.01, *:P-value < 0.05)

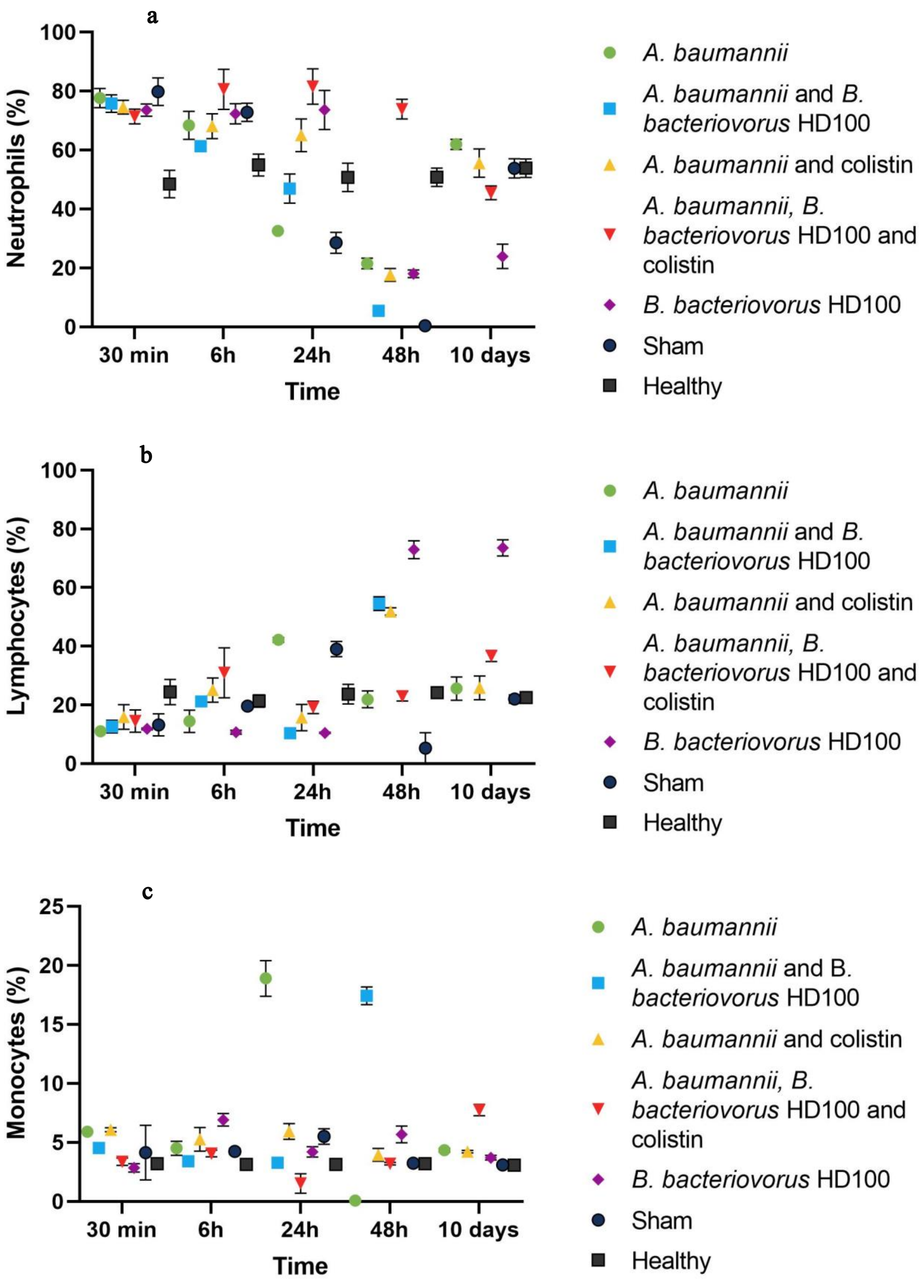

a
Neutrophils (%)
100
80
60
40
20
0
30 min
6h
24h
48h
10 days
Time
A. baumannii
A. baumannii and B. bacteriovorus HD100
A. baumannii and colistin
A. baumannii, B. bacteriovorus HD100 and colistin
B. bacteriovorus HD100
Sham
Healthy
b
Lymphocytes (%)
100
80
60
40
20
0
30 min
6h
24h
48h
10 days
Time
A. baumannii
A. baumannii and B. bacteriovorus HD100
A. baumannii and colistin
A. baumannii, B. bacteriovorus HD100 and colistin
B. bacteriovorus HD100
Sham
Healthy
c
Monocytes (%)
25
20
15
10
5
0
30 min
6h
24h
48h
10 days
Time
A. baumannii
A. baumannii and B. bacteriovorus HD100
A. baumannii and colistin
A. baumannii, B. bacteriovorus HD100 and colistin
B. bacteriovorus HD100
Sham
Healthy

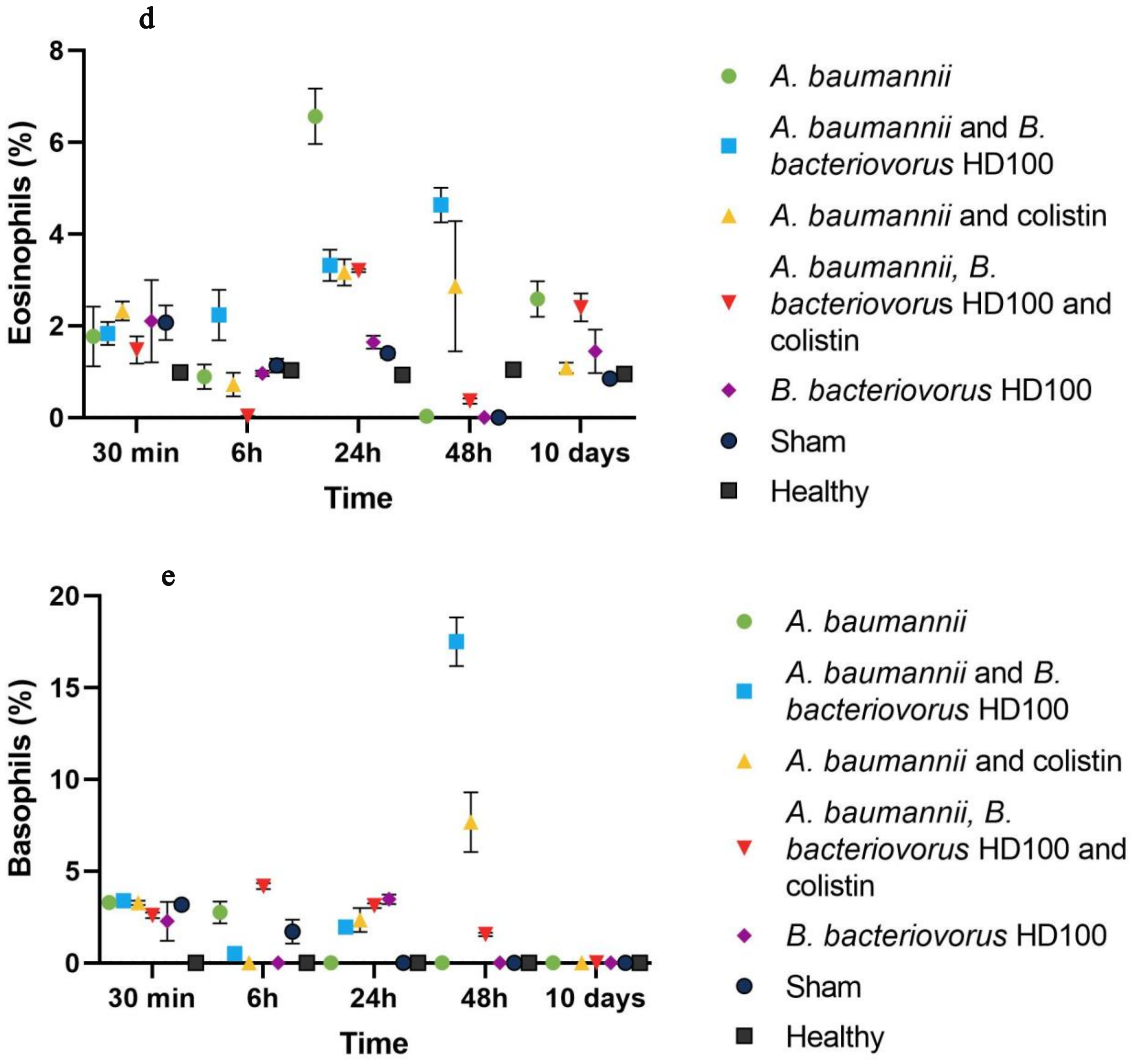

Figure A3. Neutrophils **(a)**, lymphocytes **(b)**, monocytes **(c)**, eosinophils **(d)**, and basophils **(e)** percentage for each group between 30 min and 10 days. Neutrophils counts were performed by SYSMEX XS-500i hemocytometer.
